# Platelet-derived growth factor receptor β F7 mutations result in and exacerbate the severity of vascular dysplasia in the brain arteriovenous malformation through enhancing angiogenesis

**DOI:** 10.1007/s10456-026-10044-w

**Published:** 2026-05-03

**Authors:** Alka Yadav, Leandro Barbosa Do Prado, Mustafa Mohamed, Calvin Wang, Joshua Shi, Zahra Shabani, Rich Liang, Kelly Press, Courtney Tom, Ethan A. Winkler, Hua Su

**Affiliations:** 1 Department of Anesthesia and Perioperative Care, Center for Cerebrovascular Research, University of California, San Francisco, 1001 Potrero Aven, Box 1363, San Francisco, CA 94110 USA; 2 Department of Neurosurgery, University of California, San Francisco, San Francisco, CA USA

**Keywords:** Brain arteriovenous malformation, Platelet-derived growth factor receptor-β, Pericyte, Endoglin, Alk1

## Abstract

Brain arteriovenous malformations (bAVMs) are tangles of abnormal vessels that shunt blood directly from arteries to veins. The reduction of pericytes is linked to hemorrhage in bAVMs. The Pdgfb/Pdgfrβ signaling pathway is crucial for regulating pericyte recruitment during angiogenesis. Here, we show that mice with *Pdgfrβ* F7 mutations developed cerebrovascular dysplasia in the brain’s angiogenic region, associated with increased pro-angiogenic and inflammatory signaling. Interestingly, the expression of activin receptor-like kinase 1 (Alk1) is decreased in the brain’s angiogenic region of *Pdgfrβ* F7 heterozygous mice. Overexpression of ALK1 in brain endothelial cells (ECs) reduces angiogenic signaling and the severity of vascular dysplasia in *Pdgfrβ* F7 heterozygous mice. *Pdgfrβ* F7 mutations also increased dysplastic vessels and reduced pericyte coverage in the bAVM in endoglin-deficient mice. Endoglin is an AVM causative gene. Our data indicate that *Pdgfrβ* F7 mutations result in cerebrovascular dysplasia and worsen the bAVM phenotype in endoglin mutant mice by enhancing angiogenesis and inflammation. Overexpression of ALK1 in brain ECs reduces the severity of cerebrovascular dysplasia in *Pdgfrβ* F7 heterozygous mice through downregulating angiogenic signaling.

**Supplementary Information:**

The online version contains supplementary material available at 10.1007/s10456-026-10044-w.

## Introduction

An abnormal mass of blood vessels called “nidus” is the main characteristic of brain arteriovenous malformations (bAVMs), which lead to the direct shunting of blood from arteries to veins [1]. Intracranial hemorrhage (ICH) is the most severe complication of bAVMs and the primary reason for treatment. Overall, bAVMs account for 25% of hemorrhagic stroke in children and adults < 50 years of age [2]. The cellular and/or molecular mechanisms underlying bAVM destabilization or rupture remained unclear.

Normal cerebrovascular structure and function depend on the coordinated signaling of multiple interconnected cell types, including endothelial cells (ECs), mural cells [vascular smooth muscle cells (vSMCs) and pericytes], immune cells, glial cells, and neurons [3, 4]. Pericytes are the principal mural cell population of the cerebral microvasculature, covering roughly 80–90% of the vascular wall [5]. A reduction in pericytes is associated with both vascular instability and altered hemodynamics in human bAVMs [4, 6]. We have observed a mural cell reduction in bAVM with a deficiency of activin receptor-like kinase 1 (*Alk1*, a causative gene for AVM) in mice, which is correlated with the leakage of the blood-brain barrier (BBB) [3].

Platelet-derived growth factor-B (Pdgfb)/Pdgf receptor-β (Pdgfrβ) regulates mural cell recruitment during vascular remodeling. We found that Pdgfrβ expression is reduced in *Alk1-deficient* bAVMs [3]. Thalidomide, in a class termed as immunomodulatory drugs (IMIDs), inhibits gastrointestinal bleeding and stabilizes telangiectasia vessels in patients with hereditary hemorrhagic telangiectasis (HHT) through increasing PDGFB expression [7]. Overexpression of Pdgfb reduced abnormal vessels and hemorrhage in mouse bAVMs with *Alk1* deletion [8]. These data suggest that Pdgfb/Pdgfrβ signaling is impaired in bAVM, leading to a reduction of mural cell coverage of bAVM vessels and enhancement of bAVM phenotype severity.

The genes currently known to cause human bAVM include genetic mutations of HHT causative genes: endoglin (Eng), Alk1, SMAD4 [9], and somatic activating variants in genes involved in the MAPK pathway, such as KRAS and MAP2K1, in brain ECs [10]. *Pdgfrβ* gene mutation has not been found in human bAVMs. It is unclear whether a Pdgfrβ mutation alone can cause bAVM. In this study, we tested using *Pdgfrβ *F7/WT (heterozygous) and *Pdgfrβ *F7/F7 (homozygous) mice that have all Pdgfrβ signaling disrupted [11], whether mutation of *Pdgfrβ* alone causes cerebrovascular dysplasia and increases bAVM severity in mice with *Eng* deleted in ECs, and the changes in related pathways.

## Materials and methods

### Ethics statement

All animal experimental protocols were approved by the Institutional Animal Care and Use Committee (IACUC) of the University of California, San Francisco (UCSF) and conformed to the National Institutes of Health guidelines for the care and use of laboratory animals. Veterinary care was offered by the IACUC faculty. All mice were maintained in a pathogen-free environment and were kept on a 12-hour light-dark cycle with free access to food and water. Animal experiments were conducted by certified investigators who contributed to the study.

### Animals

Five groups of 8- to 10-week-old mice: wild type (WT) mice, *Pdgfrβ F7/WT* (heterozygous) and *Pdgfrβ F7/F7* (homozygous) mice that have Pdgfrβ signaling disrupted [11], *Pdgfb*icreER;*Eng*^f/f^ mice that have *Pdgfb* promoter driving, tamoxifen (TM) inducible cre expression in ECs and a floxed *Eng* gene [12]; and *Pdgfb*icreER;*Eng*^f/f^ ;*Pdgfrβ F7/WT* mice, were used. A near equal number of male and female mice were included to exclude gender bias.

### Induction of brain angiogenesis and bAVM through stereotactic injection of viral vectors and intravenous injection of AAV vector

Brain angiogenesis was induced by stereotactic injection of an adeno-associated viral vector (AAV) expressing vascular endothelial growth factor [AAV1-VEGF, 2 × 10^9^ virus genomes (vgs)] as described in our previous papers [13] and illustrated in Supplementary Fig. 1. In brief, the AAV1-VEGF was injected into the right basal ganglia (2 mm lateral to the sagittal suture, 1 mm posterior to the coronal suture, and 3 mm under the brain surface). *Pdgfb*icreER;*Eng*^f/f^ and *Pdgfb*icreER;*Eng*^f/f^;*Pdgfrβ *F7/WT were treated with TM (2.5 mg/25 g of mouse body weight) for 3 consecutive days starting from the day of AAV1-VEGF injection to delete *Eng* in ECs and to induce bAVM. Brain samples were collected 8 weeks (Day 56) after model induction (Supplementary Fig. 2).

AAV.cc84-ALK1 (1 × 10^11^ vgs), a vector that expresses human ALK1 in brain ECs after intravenous injection [13], was delivered via the tail vein one day before the intracerebral injection of AAV1-VEGF. Control mice were injected with AAV.cc84-RFP (1 × 10^11^ vgs) (Supplementary Fig. 2) [13].

### Immunofluorescence staining

Brains were sectioned into 20 μm-thick sections using a Leica CM1950 Cryostat (Leica Microsystems, Wetzlar, Germany). Two coronal sections per mouse, about 0.5 mm rostral and 0.5 mm caudal to the virus injection site were incubated with the primary antibodies at 4 °C overnight: Rat anti-CD31 antibody (1:100, Cat #SC-18916, Santa Cruz Biotechnology, Santa Cruz, CA) to stain the ECs, Goat anti-CD13 (1:100, Cat # AF2335, R&D Systems, Minneapolis, MN) antibody to stain pericytes, and rabbit anti-mouse α-smooth muscle actin (α-SMA) antibody (1:400, Cat #A2547, Sigma, St Louis, MO) to stain vSMCs. A donkey anti-rat antibody conjugated with Alexa Fluor 488-conjugated (1:100, Cat # A-21208), donkey anti-goat antibody conjugated with Alexa Fluor 594 (1:300, Cat #A-11058), donkey anti-rabbit antibody conjugated with Alexa Fluor 555 (1:400, Cat #A-31572, Thermo Fisher Scientific, Waltham, MA), were used as the secondary antibodies to visualize positive stains. Sections were mounted with Vectashield antifade DAPI containing mounting medium (Cat #H-1200, Vector Laboratories, Burlingame, CA).

Three images were taken from each section (right and left of, and below the center of the angiogenic region. Supplementary Fig. 1) under a 20X objective lens (Leica MZFL III microscope, Leica Microsystem, Bannockburn, IL) for quantification. Thus, six 20X fields per mouse were used for quantifying vascular density and vascular pericyte coverage using NIH Image 1.63 software. Dysplastic vessels and vSMA-negative vessels were counted manually. Dysplasia index (the number of vessels with a lumen diameter larger than 15 μm/mm^2^) was used to quantify dysplastic vessels.

### Latex perfusion

Blue latex dye (Catalog BR80B, Connecticut Valley Biological Supply Co., Southampton, MA, USA) was injected into the left ventricle of the heart. Brains were collected and fixed in 4% paraformaldehyde overnight and then dehydrated in a methanol series, cleared in organic solvent (benzyl benzoate/benzyl alcohol, 1:1; Sigma-Aldrich), and imaged under a light microscope [14]. Due to the particle size, after the intra-left cardiac ventricle injection, the latex dye enters the veins only in the presence of an arteriovenous (AV) shunt.

### Prussian blue staining

Two sections per brain, about 0.5 mm rostral and 0.5 mm caudal to the virus injection site, were used to detect iron deposition using an Iron Stain Kit (Sigma-Aldrich, St. Louis, MO) according to the manufacturer’s instructions. The positively stained areas (blue) on the sections were quantified using NIH Image 1.63 software.

### RNA isolation, RNA sequencing (RNAseq), and quantitative reverse transcriptase polymerase chain reaction (qRT-PCR)

Total RNA was isolated and purified from 2 mm³ tissues around the AAV1-VEGF injected sites (located by needle holes caused by injecting AAV1-VEGF or the makers used for injection to estimate the injection site, 2 mm lateral to the sagittal suture and 1 mm posterior to the coronal suture [13], Supplementary Fig. 1) using TRIzol (Invitrogen, Carlsbad, CA), followed by RNeasy (QIAGEN, Germantown, MD) binding and quantified by a NanoDrop™ Lite (ThermoFisher Scientific, Waltham, MA, USA). A 0.2 µg RNA from each sample was sent to Novogene Co. (Sacramento, CA) for RNAseq. The data were analyzed by Novogene Co. using the company’s standard protocol (Supplemental file S1).

Differential expression analysis was used to compare the differences in gene expression across the experimental groups and to detect changes in gene expression predominantly seen in specific experimental groups. Genes with an adjusted p-value < = 0.05 found by DEseq2 were assigned as differentially expressed. Gene Ontology (GO) and Kyoto Encyclopedia of Genes and Genomes (KEGG) analyses were used to detect the molecular and signaling pathway changes among groups.

For qRT-PCR, complementary DNA (cDNA) was prepared from 2 µg total RNA using the SuperScript III First-Strand Synthesis System (Invitrogen, Carlsbad, CA). The TaqMan primers/probs, Vegfa (Catalog Mm01281449), Alk1 (Catalog Mm00437432_m1), Kdr (Mm01222421), and Akt3 (Mm01311133_m1, Thermo Fisher Scientific) were used to analyze gene expression. PCR was performed using cDNA 10 ng, 50 nmol of each primer, and Taqman Fast advanced Master Mix (Catalog number 4444963, Thermo Fisher Scientific) in 20 µl reactions following the manufacturer’s protocol on an Agilent Mx3005P Real-Time PCR system (Agilent Technologies, Santa Clara, CA). Data were normalized using endogenous control HPRT (Mm01545399_m1) or GAPDH (Catalog Mm02758991_g1, Thermo Fisher Scientific).

### Statistics

Sample sizes were calculated based on prior data with the following assumptions: α = 0.05, β = 0.2 (power 80%). For quantification, sections were randomized and analyzed by two researchers blinded to the experimental groups. Inter-observer discrepancy was controlled within one standard deviation. Data are presented as mean ± standard deviation (SD). Non-normally distributed data were log-transformed before analysis. All data were analyzed using a t-test for two-sample comparison or one-way ANOVA for multiple-sample comparison, followed by Tukey’s multiple comparisons using GraphPad Prism 10 software. A *p*-value < 0.05 was significant. Sample sizes (animal numbers) are indicated in figure legends.

## Results

1. *Pdgfrβ* F7 mutations induced pericyte loss and cerebrovascular dysplasia in mice.

**Fig. 1:**
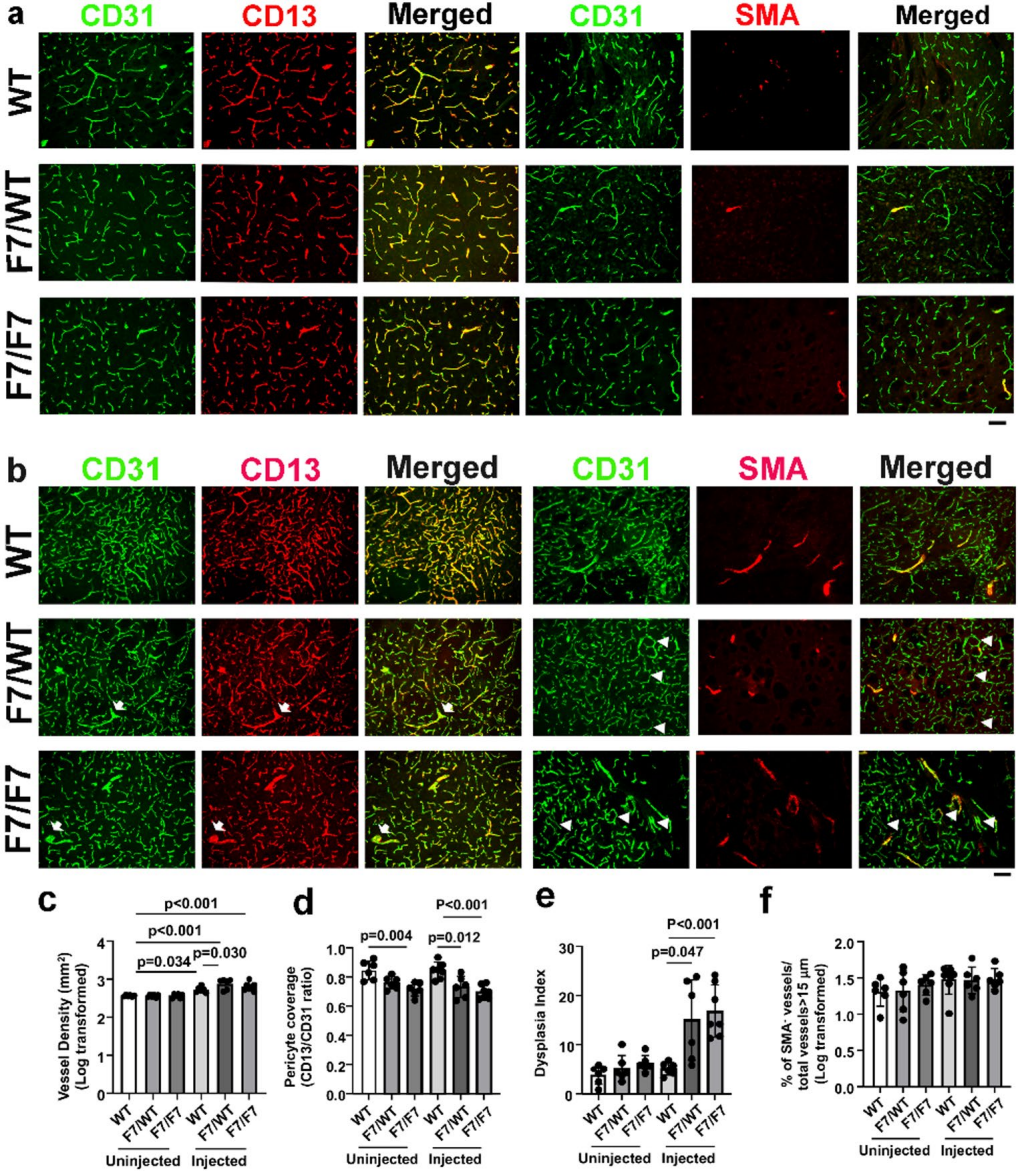
*Pdgfrβ F7* mutations induced pericyte loss and cerebrovascular dysplasia in mice. **a**. Representative images of brain sections collected from mice that have not received AAV1-VEGF injections. ECs were stained green. Pericytes and vSMCs (SMA) were stained red. Scale bar = 50 μm. **b.** Representative images of brain sections collected from mice that received AAV-VEGF injections. ECs were stained green. Pericytes and vSMCs (SMA) were stained red. Arrows indicate abnormal vessels. Arrowheads indicated vSMC-negative vessels with lumens larger than 15 μm. Scale bar = 50 μm. c-f are the quantification of **v**ascular density (**c**), pericyte coverage (**d**), dysplasia index (**d**), and % of SMA^−^ vessels/total vessels > 15 μm (**e**). F7/WT: *Pdgfrβ* F7 heterozygous mutant mice; F7/F7: *Pdgfrβ F7* homozygous mutant mice. *n* = 6–7

To test if mutations of *Pdgfrβ* alone cause cerebrovascular dysplasia, we induced brain focal angiogenesis in WT, *Pdgfrβ F7/WT*, and *Pdgfrβ F7/F7* mice and analyzed vascular mural cell-coverage, vessel density, and dysplasia index on brain sections co-stained with CD31 and CD13 antibodies, and CD31 and α-SMA antibodies. As control, no VEGF-stimulated brains from WT, *Pdgfrβ F7/WT*, and *Pdgfrβ F7/F7* mice were collected and analyzed similarly. In addition to the reduction of pericyte coverage of *Pdgfrβ F7/F7* mice compared to WT mice (*p* = 0.004, Fig. 1a & d), no other significant vascular abnormality was detected in *Pdgfrβ F7/WT*, and *Pdgfrβ F7/F7* mice without angiogenic stimulation (Fig. 1a, c, e & f).

**Fig. 2:**
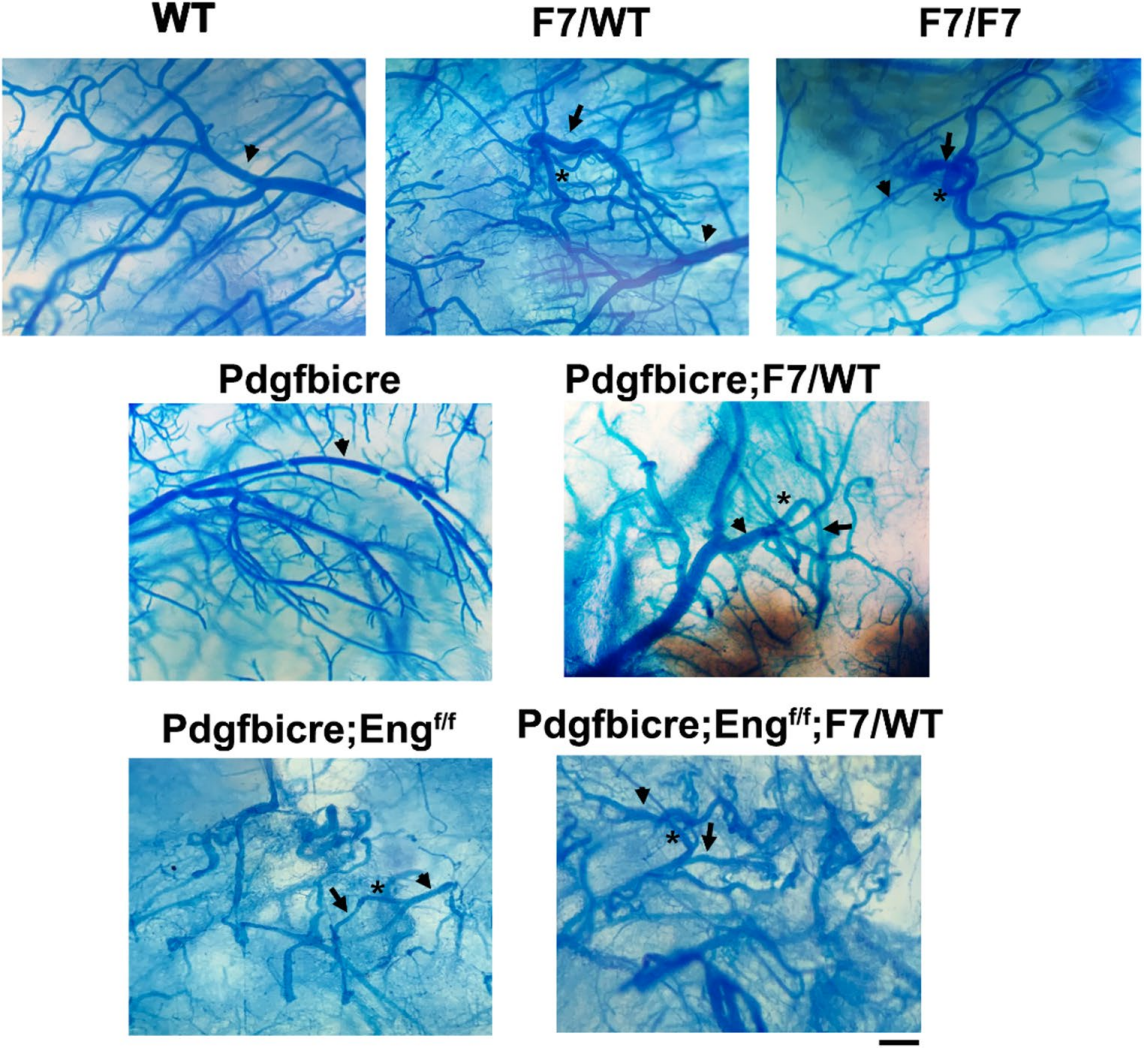
Arteriovenous (AV) shunts in the brain of *Pdgfrβ F7/WT*, *Pdgfrβ F7/F7*, *Eng*^f/f^ *and Eng*^f/f^;*Pdgfrβ F7/WT* mice. Brain vessels were cast with latex dye (blue). The particles in the latex dye are too large to pass through a capillary. Latex dye is present in the veins after being injected into the left cardiac ventricle only when in the presence of an AV shunt. Arrowheads indicate arteries. Arrows indicate veins and stars indicate AV shunts. Scale bar: 500 μm. WT: wild-type mice; F7/WT: *Pdgfrβ F7* heterozygous mice; F7/F7: *Pdgfrβ F7* homozygous mice. *n* = 3 for WT, Pdgfrbicre and Pdgfrbicre; *F7/WT* groups. *n* = 4 for *Pdgfrβ F7/WT*, *Pdgfrβ F7/F7*, *Pdgfbicre*; *Eng*^f/f^ and *Pdgfbicre*; *Eng*^f/f^;*PdgfrβF7/WT* groups

After VEGF stimulation, WT, *Pdgfrβ F7/WT*, and *Pdgfrβ F7/F7* mice had higher vessel density than unstimulated WT mice (Fig. 1a and c). *Pdgfrβ F7/WT* mice also had higher vessel density than WT mice (*p* = 0.030, Fig. 1c). *Pdgfrβ F7/WT* (*p* = 0.012) and *Pdgfrβ F7/F7* (*p* < 0.001) had lower pericyte coverage than WT mice (Fig. 1d). The pericyte coverages in *Pdgfrβ F7/WT* and *Pdgfrβ F7/F7* were similar (*p* = 0.863, Fig. 1c). *Pdgfrβ F7/WT* (*p* = 0.047) and *Pdgfrβ F7/F7* (*p* < 0.001) had more dysplastic vessels than WT mice (Fig. 1e). The number of SMA^−^ vessels were similar among all groups (Fig. 1f). *Pdgfrβ* F7 mutations also leads to hemorrhage in the brain angiogenic region (Suppl Fig. 3). We have observed AV shunts in the brain angiogenic region of *Pdgfrβ* F7/WT and *Pdgfrβ* F7/F7 mice (Fig. 2) because latex dye is present in the veins. No AV shunt was detected in the brain angiogenic region of WT and *Pdgfb*icre mice.

2. *Pdgfrβ F7* mutations reduced vascular pericyte coverage, increased dysplastic vessels in bAVMs of *Eng* EC deleted mice.

**Fig. 3:**
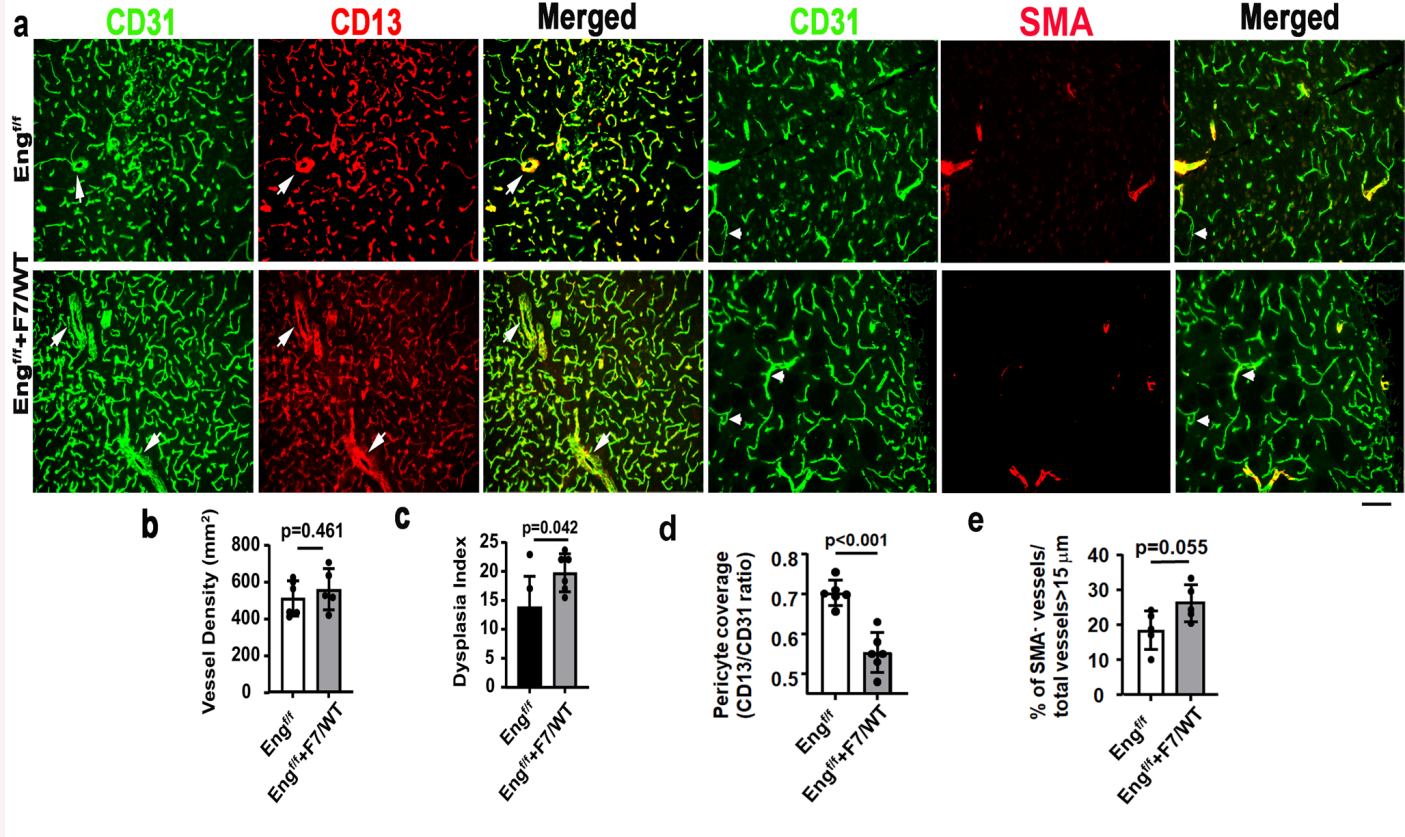
*Pdgfrβ F7* heterozygous mutations increased dysplastic vessels and reduced vascular pericyte coverage in the bAVMs of *Eng* mutant mice. **a.** Representative images of brain sections. ECs were stained green. Pericytes and vSMCs were stained red. Arrows indicate abnormal vessels. Arrowheads indicate SMA-negative vessels. Scale bar = 50 μm. **b**, **c**,** d**, and **e** are quantifications of vessel density **(b)**, dysplasia index **(c)**, Pericyte coverage **(d)** and % of SMA^−^ vessels among vessels > 15 μm **(e)**. Eng^f/f^: *Pdgfb*icreER;*Eng*^f/f^ mice; *Eng*^f/f^+F7/WT: *Pdgfbicre*ER;*Eng*^f/f^;*Pdgfrβ F7/WT* mice. *n* = 5–6

To test whether the mutations of *Pdgfrβ* enhance the severity in the bAVM of *Eng* EC-deleted mice, we induced bAVMs in *Pdgfb*icreER;*Eng*^f/f^ and *Pdgfb*icreER;*Eng*^f/f^;*Pdgfrβ F7/WT* mice and quantified vascular mural cell-coverage and the dysplasia index on brain sections stained with CD31 and CD13 antibodies, and CD31 and α-SMA antibodies (Fig. 3a). We found that compared to *Pdgfbicre*ER;*Eng*^f/f^ mice, *Pdgfbicre*ER;*Eng*^f/f^,*Pdgfrβ F7/WT* mice had more dysplastic vessels (*p* = 0.042, Fig. 3c**)** and less vascular pericyte coverage (*p* < 0.001, Fig. 3d). *Pdgfrβ* heterozygous mutations did not alter the vessel density (*p* = 0.461) and vSMC coverage (*p* = 0.055, Figs. 3b and e) in *Eng*-deficient bAVM. Microhemorrhages were detected in the bAVM lesions of *Pdgfbicre*ER;*Eng*^f/f^ mice and *Pdgfbicre*ER;*Pdgfrβ F7/WT* mice. *Pdgfrβ F7* heterozygous mutations did not further increase hemorrhage in the bAVM of *Pdgfbicre*ER;*Eng*^f/f^ mice (Suppl Fig. 3). More AV shunts were presented in latex perfused *Pdgfbicre*ER;*Eng*^f/f^;*Pdgfrβ F7/WT* mice than *Pdgfbicre*ER;*Eng*^f/f^ mice (Fig. 2).

3. *Pdgfrβ F7* heterozygous mutations increased the pro-angiogenic and pro-inflammation signaling in the brain angiogenic regions and bAVMs of *Eng* mutant mice.

**Fig. 4:**
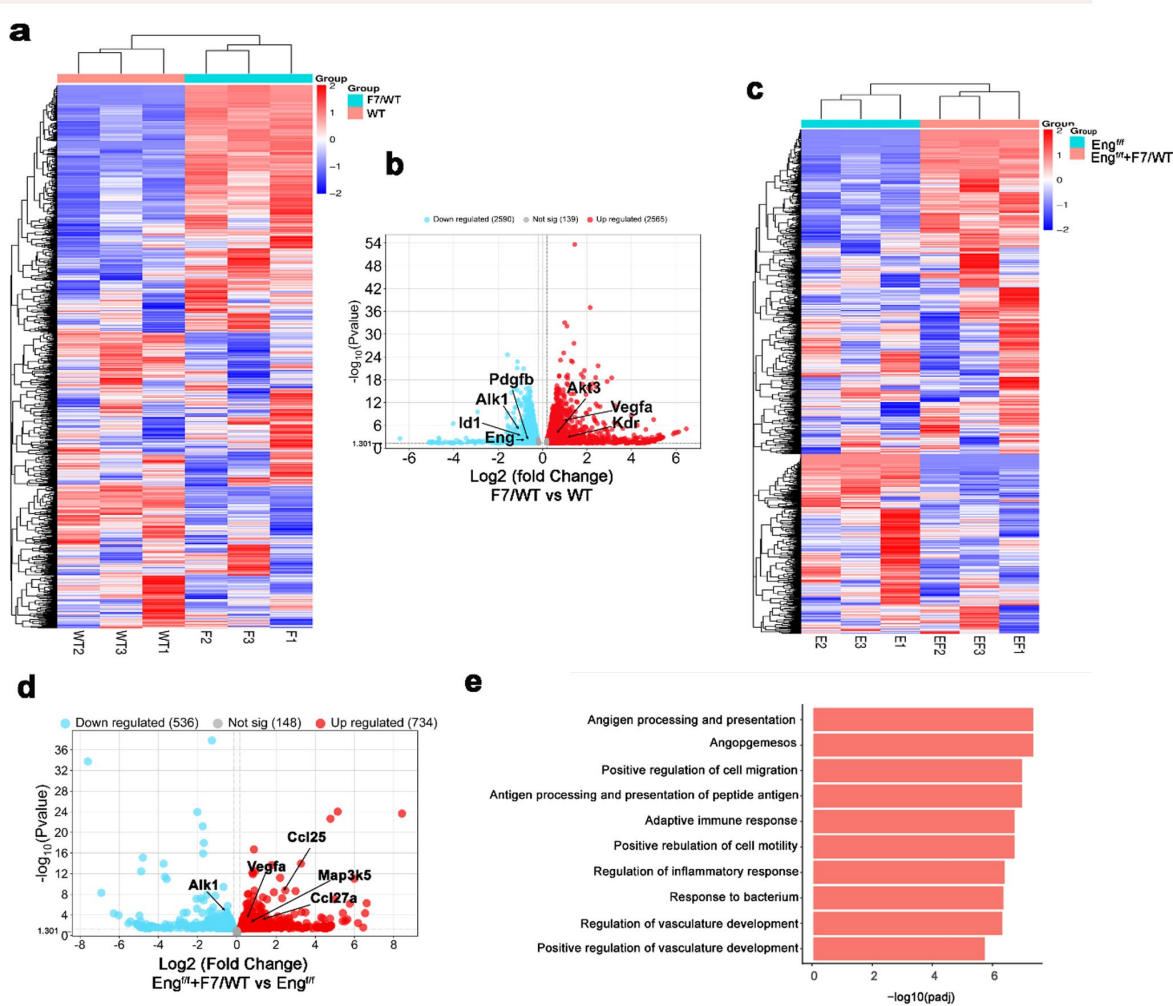
*Pdgfrβ F7* mutations increased the expression of pro-angiogenic and pro-inflammatory gene expression in the brain angiogenic region and bAVM of *Eng mutant* mice. **a** Heatmap showing differential expression of genes in the brain angiogenic regions of WT mice (WT1, WT2, WT3) and *Pdgfrβ F7/WT* mice (F1, F2, F3). **b** Volcano plots showing upregulated (red) and downregulated (blue) genes in the brain angiogenic regions of *Pdgfrβ F7/WT* mice compared to WT mice. **c** Heatmap showing differential expression of genes in the bAVMs of *Pdgrb*icreER;*Eng*^f/f^ mice (E1, E2, E3) and *Pdgrb*icreER;*Eng*^f/f^;*Pdgfrβ F7/WT* mice (EF1, EF2, and EF3). **d** Volcano plots showing upregulated (red) and downregulated (blue) genes induced by *Pdgfrβ* heterozygous (F7/WT) mutant in the bAVMs of *Pdgrb*icreER;*Eng*^f/f^ mice. Eng^f/f^+F7/WT: *Pdgrb*icreER;*Eng*^f/f^;*Pdgfrβ F7/WT* mice; Eng^f/f^: *Pdgrb*icreER;*Eng*^f/f^ mice. **e** Top changed biological pathways induced by *Pdgfrβ F7/WT* mutant in the bAVMs of *Pdgrb*icreER;*Eng*^f/f^ mice identified by GO analysis. The cutoff threshold for b and d is 1.301

**Fig. 5:**
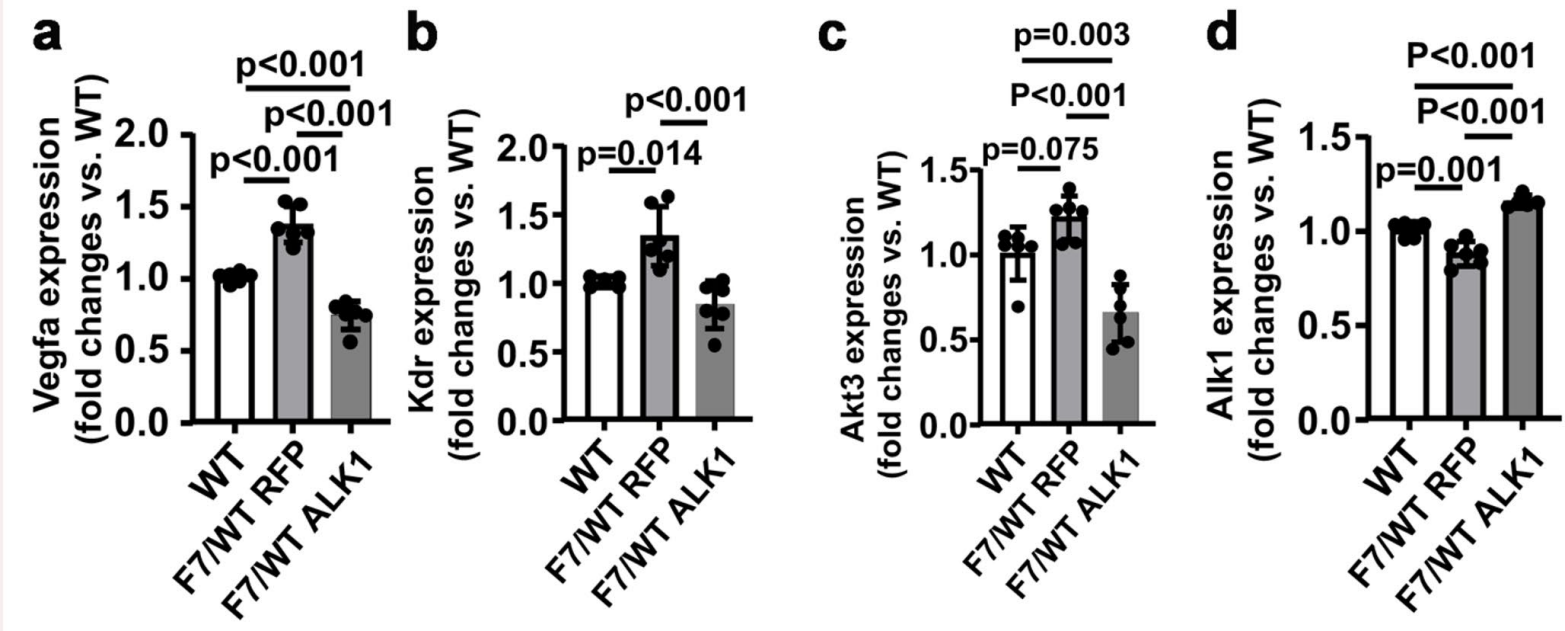
*Pdgfrβ F7* heterozygous mutations upregulated pro-angiogenic gene expression and downregulated Alk1 expression; overexpression of ALK1 in brain ECs reversed the gene expression profiles. Quantification of Vegfa (**a**), Kdr (**b**), Akt3 (**c**), and Alk1 (**d**) expression. WT: wild type mice; F7/WT RFP; *Pdgfrβ F7* heterozygous mice treated with AAV.cc84-RFP; F7/WT ALK1: *Pdgfrβ F7* heterozygous mice treated with AAV.cc84-ALK1. *N* = 5–6

RNAseq was performed to investigate the influence of *pdgfrβ F7* mutations in RNA transcriptomics in the brain angiogenic region induced by stereotactic injection of AAV1-VEGF (Suppl Fig. 1) of WT mice, Pdgfrβ F7 heterozygous mice, and bAVM of *Pdgfb*icreER;*Eng*^f/f^ and PdgfbicreER:Engf/f;Pdgfrβ F7/WT mice. Differential analysis showed a distinctive gene expression profile in the brain angiogenic region of WT mice and *pdgfrβ F7/WT* mutant mice (Fig. 4a). *Pdgfrβ F7* heterozygous mutations upregulated the expression of pro-angiogenic genes, Vegfa, Kdr, and Akt3, and downregulated genes related to pericyte recruitment and *Tgfβ1* signaling, *Pdgfb*, *Alk1*, *Eng*, and *Id1* (Figs. 4b and 5 & Suppl Table 1).

*Pdgfrβ F7* heterozygous mutations also induced distinctive changes in gene expression (Fig. 4c) and transcriptional changes in the bAVM of *Pdgfb*icreER;*Eng*^f/f^ mice, increased pro-angiogenic and pro-inflammatory gene expression and signaling (Fig. 4d & e, Suppl Table 2).

The top-upregulated genes and down-regulated genes in the comparisons of *Pdgfrβ F7/WT* versus WT and *Pdgfb*icreER;*Eng*^f/f^;*Pdgfrβ F7/WT* versus *Pdgfb*icreER;*Eng*^f/f^ are listed in Suppl Tables 3, 4, 5 & 6. Since we did bulk RNAseq, many of those genes are not related to vascular stability. However, Go and KEGG analyses identified several pathways involved in vascular functions and inflammation were upregulated in *Pdgfrβ F7/WT* mice versus WT mice, including PI3K (GO, Adj. *p* = 0.008), TOR signaling (GO, Adj. *p* = 0.037), MAP kinase activity (GO, Adj. *p* = 0.043), regulation of EC migration (GO, Adj. *p* = 0.043), IL17 signaling pathways (KEGG, Adj. *p* = 0.016) and Ras signaling pathway (KEGG, Adj. *p* = 0.019), and in bAVMs of of *Pdgfb*icreER:*Eng*^*f/f*^;*Pdgfrβ F7/WT* mice versus *Pdgfb*icreER:*Eng*^*f/f*^ mice (Fig. 4e).

Together, these findings suggest that the *Pdgfrβ F7* heterozygous mutations enhance angiogenic and inflammatory signaling pathways in the brain angiogenic region and *Eng*-deficient bAVMs, leading to a more severe bAVM phenotype.

4. Overexpression of ALK1 in brain ECs reduced the severity of cerebrovascular dysplasia in *Pdgfrβ F7/WT* mice.

**Fig. 6:**
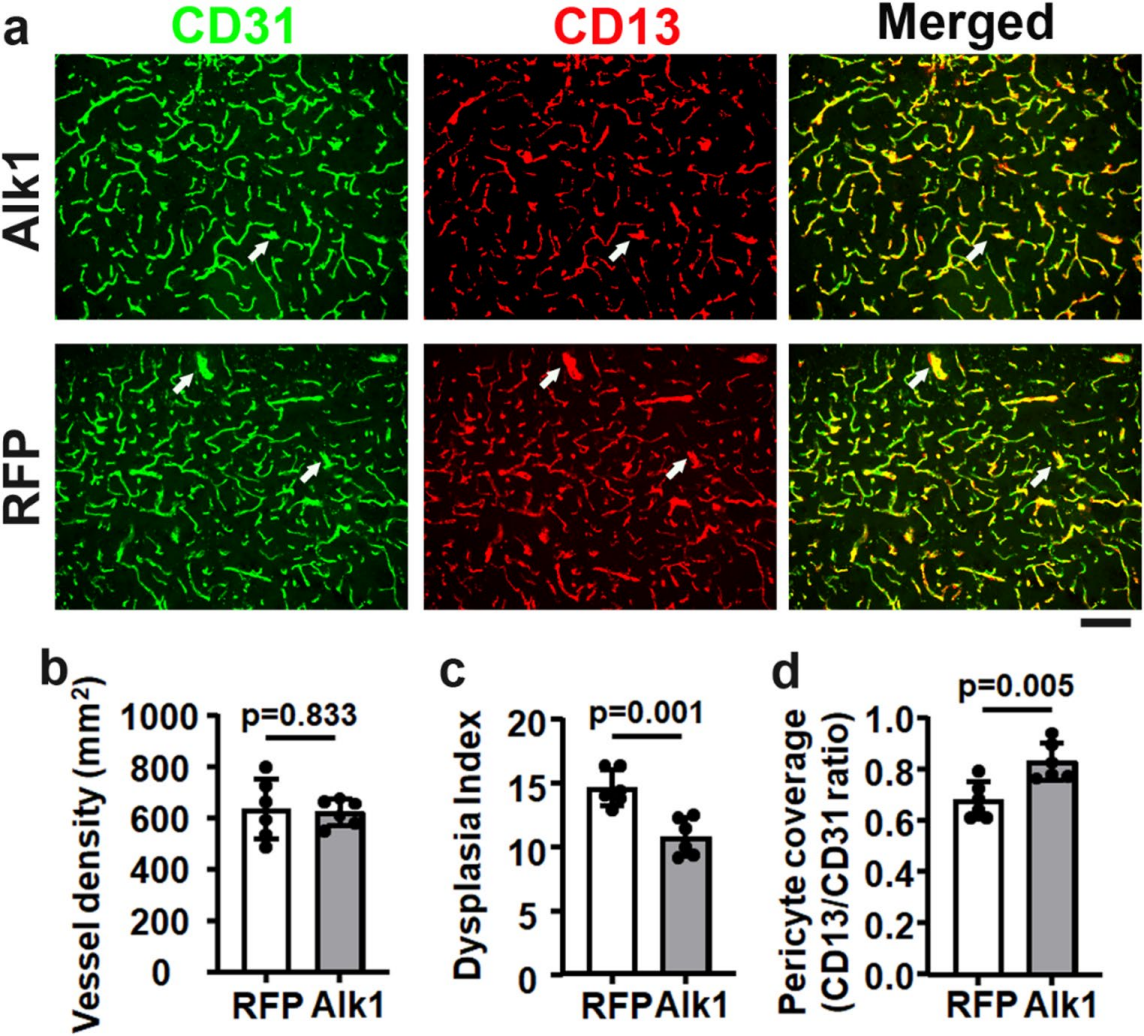
Overexpression of ALK1 in the brain ECs reduced the severity of cerebrovascular dysplasia in *Pdgfrβ F7/WT* mice. **a.** Representative images of brain sections. ECs were stained green. Pericytes were stained red. Arrows indicate dysplastic vessels. Scale bar = 50 μm. **b–d** are the quantification of **v**ascular density (**b**), dysplasia index (**c**), and pericyte coverage (**d**). *n* = 6. Alk1: AAV.cc84-ALK1 treated mice; RFP: AAV.cc84-RFP treated mice

Interestingly, *Pdgfrβ F7* heterozygous mutations reduced Alk1 expression in the brain angiogenic region. Since the mutation of the *Alk1* gene in ECs leads to AVM development [15], we tested whether overexpression of ALK1 (human gene) in brain ECs reduces the severity of cerebrovascular dysplasia in *Pdgfrβ F7/WT* mice. We used an AAV vector, AAV.cc84, which we have developed, that can transduce brain ECs through intravenous injection [13] to deliver the ALK1 gene into the brain ECs of *Pdgfrβ F7/WT* mice. Intravenous injections of AAV.cc84-ALK1 and AAV.cc84-RFP did not alter the cerebrovascular structure of WT mice [15]. We found in this study that overexpression of ALK1 in brain ECs reduced the dysplasia index and increased pericyte coverage in the brain angiogenic region of *Pdgfrβ F7/WT* mice (Fig. 6). Overexpression of ALK1 in brain ECs has also reduced pro-angiogenic gene expression (Fig. 5).

These data indicated that overexpression of ALK1 in brain ECs of *Pdgfrβ F7/WT* mice reduces the severity of cerebrovascular dysplasia through downregulation of angiogenesis.

## Discussion

In this study, we found that inactivation of the main downstream signaling pathways of Pdgfrβ via the F7 mutations triggers cerebrovascular dysplasia following angiogenic stimulation. *Pdgfrβ F7* mutations also enhanced the severity of bAVMs in *Eng* mutant mice through upregulating pro-angiogenic and pro-inflammatory genes and pathways. *Pdgfrβ F7* heterozygous mutations reduced the expression of several genes involved in Tgfβ1 signaling, including *Alk1* and *Eng*, both are AVM causative genes, in the brain angiogenic region. Overexpression of the human *ALK1* gene in brain ECs reduced the severity of cerebrovascular dysplasia in *Pdgfrβ F7/WT* mice, suggesting that downregulation of Tgfβ1 signaling is one of the mechanisms underlying the cerebrovascular dysplasia induced by *Pdgfrβ F7* mutations.

Pdgfrβ is expressed in multiple cell types, including pericytes, vSMCs, and neurons [16]. Its ligand, Pdgfb, is secreted from the ECs of angiogenic sprouts, where it works as an attractant for pericytes. Homozygous deletion of *Pdgfb* or *Pdgfrβ* in rodents results in high embryonic mortality due to widespread hemorrhage [17]. Disruption of Pdgfb/Pdgfrβ signaling also causes excessive vascular abnormalities and microaneurysms [18]. *Pdgfrβ F7* mutant mice have less pericyte coverage in the brain and spinal cord of adult 6–8-month-old mice [5]. Consistent with these findings, we showed a significant reduction of pericyte coverage, abnormal vessel morphology, and microhemorrhages in the brain angiogenic region of *Pdgfrβ F7/WT* and *Pdgfrβ F7/F7* mice. In addition, we showed that *Pdgfrβ* F7 heterozygous mutations upregulates the expression of pro-angiogenic genes and downregulates the expression of genes related to Tgfβ1 signaling in the brain angiogenic region. Interestingly, PI3K/mTOR signaling was upregulated in the brain angiogenic region of *Pdgfrβ F7/WT* mice, despite Pdgfrβ-mediated PI3K signaling being inactivated in *Pdgfrβ F7* mutant mice [11]. The potential explanations are (1) our data were collected from bulk RNAseq; the upregulated PI3K signaling could be from cells that do not express Pdgfrβ; (2) compensatory endothelial response to reduced pericyte coverage and vessel instability; (3) in addition to Pdgfrβ, PI3K/mTOR signaling can be activated by other pathways, such as loss of the tumor suppressor PTEN, activation of receptor tyrosine kinases (RTKs), etc [19]. and Pdgfrα [20]; and (4) the samples used for RNAseq were from *Pdgfrβ F7* heterozygous (*Pdgfrβ F7/WT*) mice; the remaining WT *Pdgfrβ* allele can also upregulate PI3K pathway upon angiogenic stimulation.

Abnormal vascular remodeling and vascular instability are associated with bAVM development and progression [4]. Abnormal expressions of Pdgfb and Pdgfrβ has been detected in bAVMs in humans and rodents [3, 6]. Pdgfrβ expression is reduced in the bAVM lesions of *Alk1* mutant mice, which is associated with a reduction of mural cell coverage [3]. We showed that Alk1 expression is reduced in the brain angiogenic region of *Pdgfrβ F7/WT* mice, which is associated with the development of cerebrovascular dysplasia. Overexpression of human ALK1 in brain ECs reduced the severity of vascular dysplasia in the brain angiogenic region of *Pdgfrβ F7/WT* mice. These findings suggest a possible crosstalk between ALK1 and PDGFRβ. However, direct binding between these two receptors has not been identified. Currently, it is understood that ALK1 activity in ECs regulates PDGFB production, which subsequently signals through PDGFRβ in pericytes. The mechanism by which PDGFRβ affects ALK1 expression or function remains unclear and needs to be studied further.

Reduction of mural cell coverage in the bAVM vessels in mouse models and human specimens is associated with the reduction of Pdgfb and Pdgfrβ protein levels in bAVM lesions and bAVM bleeding [3, 6]. The role of PDGFB/PDGFRβ signaling in human bAVM pathogenesis has not been studied fully. In this study, we tested the influence of *Pdgfrβ F7* mutations on bAVMs using a mouse bAVM model we have developed by deletion of *Eng* in ECs plus focal angiogenic stimulation [14]. We found that *Pdgfrβ F7* heterozygous mutations increased the number of abnormal vessels and reduced vascular pericyte coverage in the bAVMs of *Eng*-deficient mice, which is associated with increased pro-angiogenic and pro-inflammatory signaling. Although *Pdgfrβ F7* heterozygous mutations increased angiogenic signaling, the vessel density was not increased in bAVM of mice with *Eng* deletion in ECs. The possible explanation is that increased angiogenic signaling does not necessarily result in higher vessel density in bAVMs, as the dysplastic remodeling involves abnormal vessel enlargement, fusion, and instability in addition to increased sprouting. Larger AVM vessels also occupy more areas and thus reduce the vessels per unit area. Hence, total vessel density may remain unchanged despite hyper-angiogenic signaling. Overall, these data indicate that Pdgfrβ plays a role in bAVM pathogenesis.

Interestingly, *Pdgfrβ F7* mutations decreased the expression of Alk1 and Eng in the brain’s angiogenic region, and Alk1 expression in the bAVMs of Eng-deficient mice, indicating a regulatory role of Pdgfrβ on Alk1 and Eng expression. To test whether reducing Alk1 expression contributes to cerebrovascular dysplasia in *Pdgfrβ F7* mutant mice, we overexpressed a human ALK1 gene in brain ECs of *Pdgfrβ F7/WT* mice. We found that overexpression of ALK1 in brain ECs reduces the expression of pro-angiogenic genes and the severity of cerebrovascular dysplasia in the brain’s angiogenic region in *Pdgfrβ F7/WT* mice.

During angiogenesis, pericyte recruitment to EC tubes is orchestrated by several signaling cascades, including PDGFB/PDGFRβ, TGFβ, NOTCH, VEGF, and angiopoietin pathways [21]. Disruption of any component of this signaling network can impair pericyte recruitment and vascular stability. In addition to reducing mural cell recruitment, the *Pdgfrβ F7* mutations induced expression of genes associated with angiogenesis and inflammation in *Eng*-deficient bAVMs. Interestingly, fewer differentially expressed genes related to vascular genesis and inflammation were detected between *Eng*^*f*/f^ deletion alone versus *Eng*^f/f^ deletion with *Pdgfrβ F7* heterozygous mice than between *Pdgfrβ F7* heterozygous mice versus WT mice, although the same threshold was used (Fig. 4b and d). It could be that altered gene expression in response to *Eng* deletion [22] and *Pdgfrβ F7* mutations are similar. Co-mutations of *Pdgfrβ* and *Eng* not only disrupts mural cell recruitment but also creates a pro-angiogenic and pro-inflammatory microenvironment that drives bAVM progression. Furthermore, chronic inflammation in this context likely acts in a feedforward loop, where increased cytokines and chemokines further reduce pericyte recruitment and exacerbate vascular instability. Taken together, our findings establish a mechanistic link between Pdgfrβ mutations and the development of vascular dysplasia, highlighting the critical interplay between pericyte recruitment, angiogenic signaling, and inflammatory processes in bAVM pathogenesis.

Notably, overexpression of ALK1 in brain ECs ameliorates the vascular abnormalities induced by *Pdgfrβ F7* mutations. Thus, ALK1 is not only a downstream effector influenced by Pdgfrβ activity but also a valuable therapeutic target for restoring vascular stability. Pharmacological agents or gene therapy approaches aimed at enhancing ALK1 expression or signaling may offer a promising strategy to mitigate bAVM severity, especially in individuals with dysfunctions of PDGFB/PDGFRβ and TGFβ pathways. Future studies should focus on elucidating the molecular crosstalk between PDGFRβ and ALK1, and on developing targeted therapies that restore balance to this signaling axis in cerebrovascular disease.

This study has several limitations. (1) CD13 was used to label pericytes. However, CD13 is also expressed by other cell type, such as microglia, macrophages, ECs [23] and vSMCs [24]. To minimize overestimating vascular pericyte coverage, we only quantified CD13-positive cells that were closely attached to the ECs on the microvasculature. The vSMCs are mostly located around large vessels. Although inclusion of a small number of vSMCs may have led to a modest overestimation of pericyte coverage, this potential bias would be expected to occur similarly across all experimental groups. Therefore, differences among groups are still correctly reflected. (2) We did bulk RNAseq instead of scRNAseq. Many of the differentially expressed genes are not related to vascular stability. However, Go and KEGG analyses identified several pathways associated with angiogenesis and inflammation that were upregulated by the *Pdgfrβ F7* heterozygous mutations in both the WT brain angiogenic regions and the bAVMs of *Eng* EC–deleted mice. Collectively, these findings indicate that *Pdgfrβ F7* heterozygous mutations enhance angiogenic and inflammatory signaling in the brain angiogenic region and in *Eng*-deficient bAVMs, contributing to a more severe bAVM phenotype. (3) In addition, bulk RNAseq cannot definitively separate cell-intrinsic transcriptional changes from shifts in cellular composition, and scRNAseq would be needed to fully resolve cell-type-specific transcriptional responses. This represents an important direction for future studies. (4) In this study, we investigated the impact of Pdgfrb deficiency on HHT bAVMs. However, HHT-associated bAVM accounts for approximately 5% of all bAVMs in patients [25, 26]. The majority of bAVMs are sporadic, caused by somatic activating mutations in the KRAS-MAPK pathway [27, 28]. Both HHT-associated and sporadic AVMs exhibit reduced pericyte coverage and vascular instability [3, 6]. Therefore, it is interesting to explore the role of Pdgfrb in sporadic bAVM in future studies.

Overall, this study shows that the *Pdgfrβ F7* mutations alone result in cerebrovascular dysplasia in the brain angiogenic region and enhance bAVM severity of mice with *Eng* deleted in ECs by increasing pro-angiogenic and pro-inflammatory signaling. Overexpression of ALK1 in brain ECs mitigated cerebrovascular dysplasia in *Pdgfrβ F7* heterozygous mice.

## Supplementary Information

Below is the link to the electronic supplementary material.

Supplementary Material 1

Supplementary Material 2

## Data Availability

The authors declare that all data supporting the findings of this study are available in the paper and its Supplementary Information.
