# Supplementary material for: Platelet-derived growth factor receptor β F7 mutations result in and exacerbate the severity of vascular dysplasia in the brain arteriovenous malformation through enhancing angiogenesis
Source: Angiogenesis. 2026 May 3;29(3):28. doi: 10.1007/s10456-026-10044-w (PMC13136212; doi:10.1007/s10456-026-10044-w)
Supplement: Supplementary file 1 — Supplementary Material 1 [file 10456_2026_10044_MOESM1_ESM.docx]

**Platelet-derived growth factor receptor** **β F7 mutations result in and exacerbate the severity of vascular dysplasia in the brain arteriovenous malformation through enhancing angiogenesis**

**Running Title:** Mutation of Pdgfrβ enhances bAVM severity

Alka Yadav^1^, Leandro Barbosa Do Prado^1^, Mustafa Mohamed^1^, Calvin Wang^1^, Joshua Shi^1^, Zahra Shabani^1^, Rich Liang^1^, Kelly Press^1^, Courtney Tom^1^, Ethan A. Winkler^2^, Hua Su^1^

^1^Center for Cerebrovascular Research, Department of Anesthesia and Perioperative Care, University of California, San Francisco, California, USA.

^2^Department of Neurosurgery, University of California, San Francisco, California, USA

**Supplementary Figures:**

**
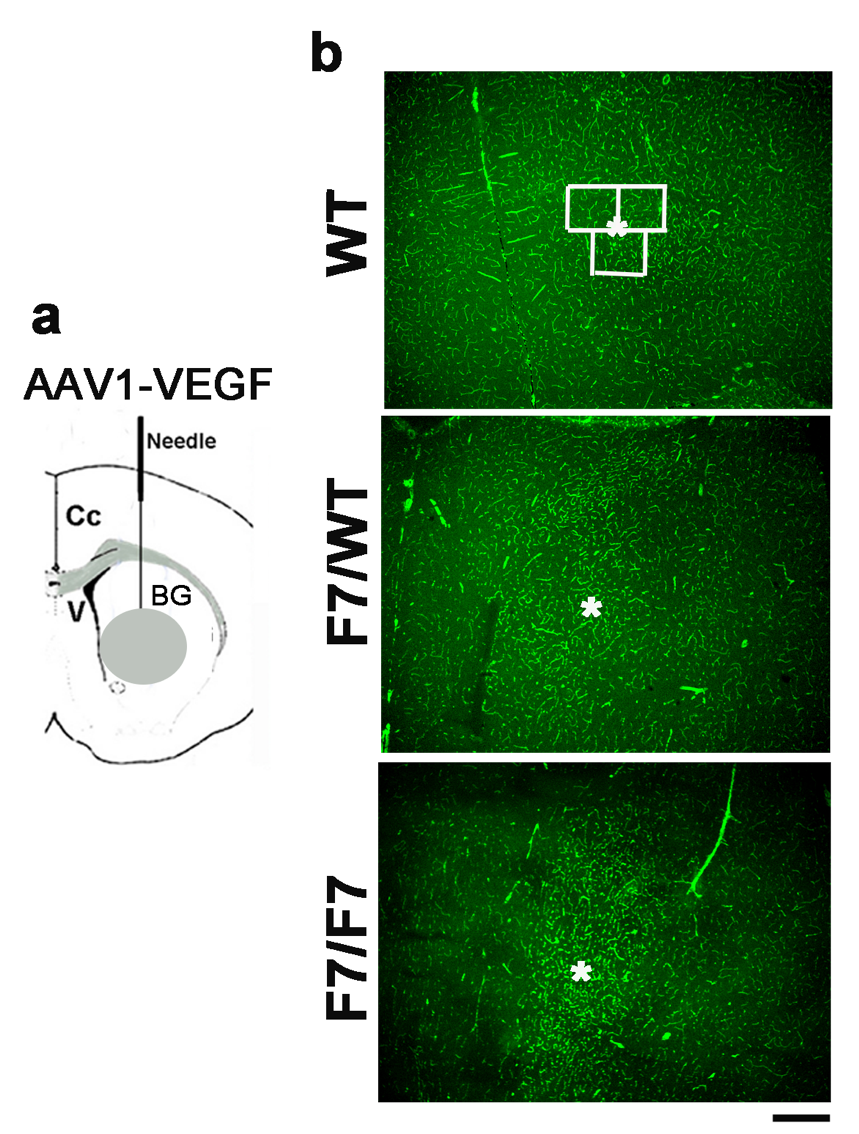
**

**Suppl Fig 1: An illustration of the AAV1-VEGF injection site, and pictures show increased vessel density around the injection sites. (a)** AAV1-VEGF was injected into the basal ganglia (BG). **(b**) Increased vessel density around the injection sites. Vessels were stained green using an anti-CD31 (a marker of endothelial cells) antibody. Scale bar:100 μm. *: center of the angiogenic area. The squares indicate the image taken area. WT: wild-type mice; F7/WT: *Pdgfrβ* *F7* heterozygous mice; F7/F7: *Pdgfrβ* *F7* heterozygous mice.

**
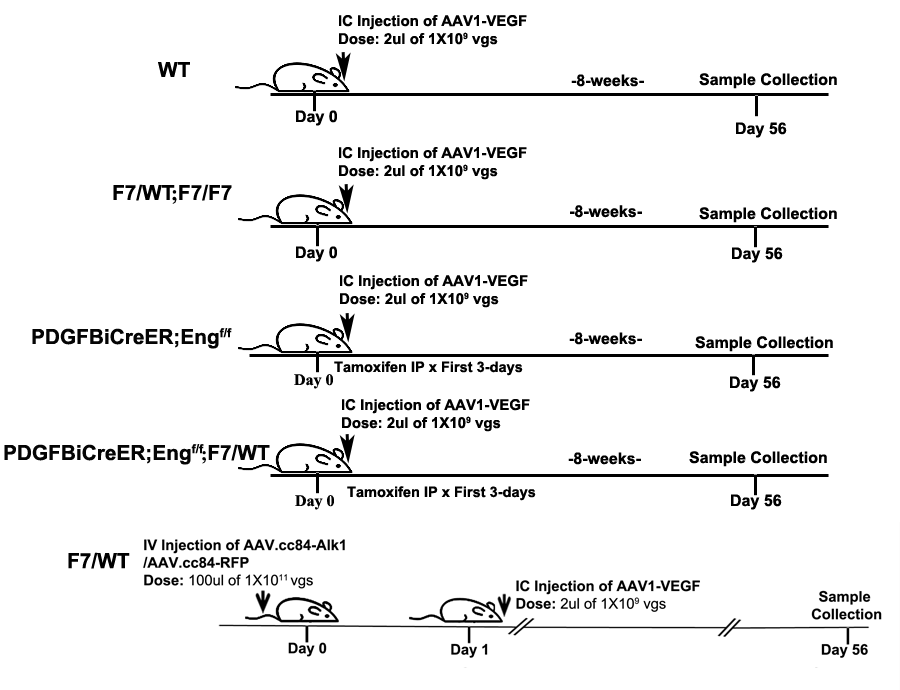
**

**Suppl Fig 2: Timeline for model induction and sample analysis.**

*
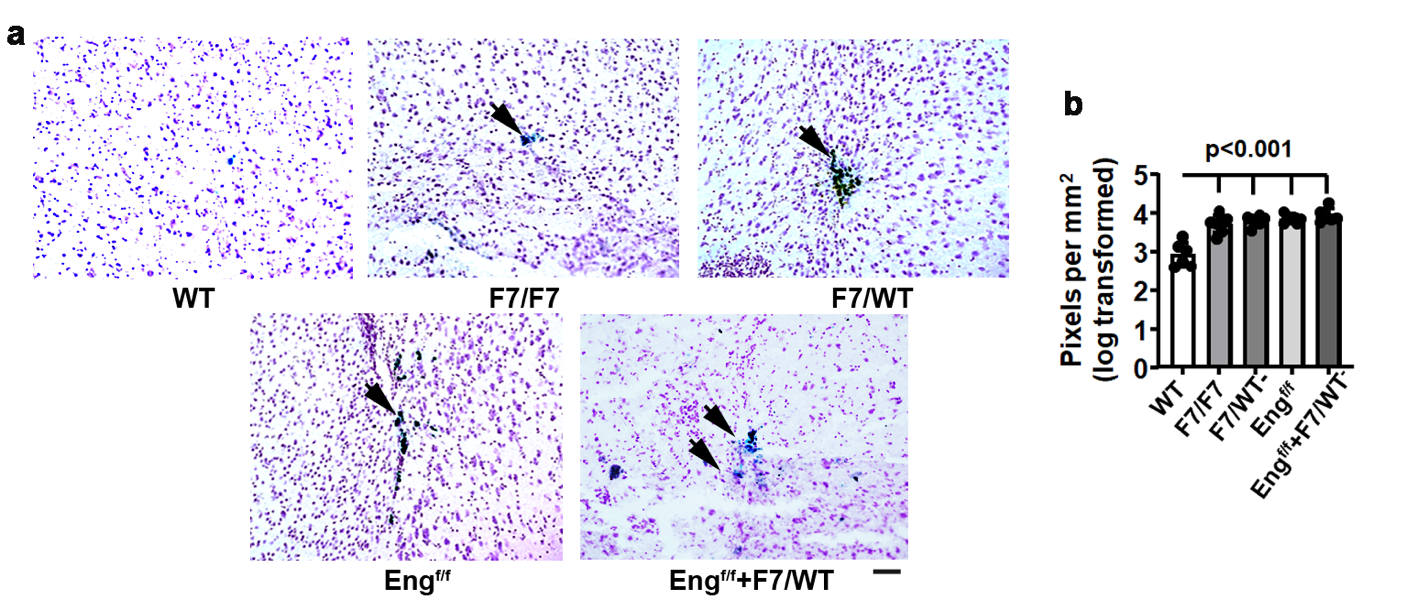
*

**Suppl Fig 3. *Pdgfrβ* *F7* mutations and *Eng* mutation cause hemorrhage in the brain. a.** Prussian-stained sections. The arrows indicate the iron-deposited area (hemorrhage). Scale bar: 50 µm. **b.** Quantification of microhemorrhage. n=6-7. F7/F7: *Pdgfrβ F7* homozygous mice; F7/WT: *Pdgfrβ F7* heterozygous mice; Eng^flf^: *Pdgfb*icreER;*Eng*^f/f^ mice; Eng^flf^+*F7/WT*: *Pdgfb*icreER;*Eng*^f/f^;*Pdgfrβ* *F7/WT* mice.

**Supplementary Tables:**

| Suppl Table 1. Key differentially expressed genes related to angiogenesis induced by *Pdgfrβ*  *F7* heterozygous mutations in the brain angiogenic region. | | | |
| --- | --- | --- | --- |
| Gene | Log2 fold-change | p-value | Adjst p-value |
| Pdgfb | -0.23 | 0.04 | 0.155 |
| Acvrl1/Alk1 | -0.53 | 0.0003 | 0.035 |
| Eng | -0.27 | 0.015 | 0.070 |
| Id1 | -0.41 | 0.028 | 0.112 |
| Vegfa | 0.61 | 6.66E-06 | 0.00013 |
| Akt3 | 0.38 | 0.0006 | 0.006 |
| Tgfβ1 | -0.66 | 0.002 | 0.0173 |
| Kdr | 0.73 | 0.002 | 0.019 |

| Suppl Table 2. Key differentially expressed genes related to angiogenesis and inflammation were upregulated by *Pdgfrβ* *F7* heterozygous mutations in the bAVMs of *Eng*-deficient mice. | | | |
| --- | --- | --- | --- |
| Gene | Log2 fold-change | p-value | Adjst p-value |
| Klf2*** | -1.6 | 1.10E-18 | 2.63E-15 |
| Ccl25** | 0.978 | 1.68E-06 | 0.0007 |
| Ccl27a** | 1.89 | 4.07E-05 | 0.010 |
| S100a9** | -3.5 | 5.41E-08 | 3.25E-05 |
| S100a8** | -3.8 | 3.62E-07 | 0.00017 |
| C1qa** | -0.7 | 5.46E-07 | 0.00024 |
| Klf4*** | -1.2 | 3.31E-06 | 0.00127 |
| Tmem181b** | -1.4 | 2.35E-05 | 0.0068 |
| Map3k5*** | 0.304 | 0.024 | 0.0552 |
| Tgfβ1*** | -0.74 | 0.0002 | 0.0368 |
| Vegfa*** | 0.3 | 0.048 | 0.6723 |
| *** Angiogenesis-related genes  ** Inflammation-related genes | | | |

| **Suppl Table 3. Top 20 upregulated genes in *Pdgfrβ* *F7/WT* mice compared to WT mice** | | | |
| --- | --- | --- | --- |
| Gene | Log2 fold-change | p-value | Adjst p-value |
| Prepl | 1.44180421 | 2.51E-54 | 4.16E-50 |
| Nos1ap | 2.13952785 | 9.72E-38 | 8.06E-34 |
| Malat1 | 0.99893733 | 9.20E-34 | 5.08E-30 |
| Glg1 | 1.09717724 | 7.69E-33 | 3.19E-29 |
| Ptprn2 | 1.41222977 | 2.55E-28 | 8.45E-25 |
| Dock3 | 0.95237973 | 8.85E-26 | 2.45E-22 |
| 2610507B11Rik | 0.80463048 | 7.04E-24 | 1.46E-20 |
| Zgpat | 1.28643258 | 1.03E-23 | 1.90E-20 |
| Atp2b3 | 1.34768571 | 2.01E-23 | 3.03E-20 |
| Gpam | 2.49661621 | 2.13E-22 | 2.94E-19 |
| Nktr** | 1.87952329 | 3.89E-21 | 4.30E-18 |
| Dgkg | 1.73961703 | 3.55E-20 | 3.68E-17 |
| Ksr2* | 1.12653769 | 5.85E-20 | 5.71E-17 |
| Kif5a | 0.75206385 | 9.07E-20 | 8.36E-17 |
| Meg3 | 0.7413981 | 1.18E-19 | 1.03E-16 |
| Arhgef11 | 1.06602774 | 1.70E-19 | 1.41E-16 |
| Usp33 | 1.8304294 | 2.12E-19 | 1.67E-16 |
| Timp3** | 1.38810133 | 2.73E-19 | 2.06E-16 |
| Tph2 | 3.10829486 | 3.04E-19 | 2.19E-16 |
| Gon4l | 1.13449975 | 3.96E-19 | 2.74E-16 |
| *Gene in MAPK/ERK cascade  **Immune and Inflammation related genes | | | |

| **Suppl Table 4. Top 20 downregulated genes in *Pdgfrβ* *F7/WT* mice compared to WT mice** | | | |
| --- | --- | --- | --- |
| Gene | Log2 fold-change | p-value | Adjst p-value |
| Rps13-ps1 | -1.5785065 | 2.75E-25 | 6.52E-22 |
| Hbb-bs | -1.1354069 | 1.74E-23 | 2.88E-20 |
| Egr4 | -1.144368 | 7.93E-22 | 1.01E-18 |
| mt-Rnr1 | -0.8539867 | 1.22E-21 | 1.45E-18 |
| Hba-a1 | -0.9582093 | 9.42E-18 | 6.01E-15 |
| Cox4i1 | -0.5920546 | 1.06E-16 | 5.33E-14 |
| Dlgap3 | -0.7268141 | 2.04E-16 | 9.51E-14 |
| Arl4d | -1.2651467 | 3.13E-16 | 1.30E-13 |
| Fosb | -1.1423912 | 4.75E-16 | 1.83E-13 |
| Dynll1 | -0.5633528 | 9.49E-16 | 3.50E-13 |
| Cst3 | -0.5434058 | 1.50E-15 | 5.29E-13 |
| Hspb1 | -1.3962618 | 2.34E-15 | 7.60E-13 |
| Pcp4 | -0.9844133 | 2.52E-15 | 8.05E-13 |
| Cnih2 | -0.6506889 | 3.61E-15 | 1.13E-12 |
| Rpl12 | -0.5548882 | 2.06E-14 | 5.34E-12 |
| mt-Rnr2 | -0.7363946 | 3.34E-14 | 8.44E-12 |
| Rpl18a | -0.5716761 | 3.36E-14 | 8.44E-12 |
| Ssbp4 | -0.8332252 | 4.55E-14 | 1.13E-11 |
| Manf | -0.8313341 | 8.72E-14 | 2.04E-11 |
| Ptms | -0.6114349 | 1.34E-13 | 3.00E-11 |

| **Suppl Table 5. Top 20 upregulated genes induced by *Pdgfrβ* *F7* heterozygous mutations in the bAVM of *Eng* EC deleted mice** | | | |
| --- | --- | --- | --- |
| Gene | Log2 fold-change | p-value | Adjst p-value |
| BC002163* | 6.2565975 | 7.42E-102 | 1.43E-97 |
| Gm11223* | 2.5975187 | 1.82E-34 | 1.16E-30 |
| 1500015A07Rik* | 2.0138274 | 1.13E-24 | 4.36E-21 |
| Hmga1b | 1.7399948 | 6.69E-22 | 1.84E-18 |
| Nudc-ps1* | 1.7191986 | 1.23E-16 | 2.37E-13 |
| Gm9008 | 4.7916849 | 7.77E-16 | 1.36E-12 |
| Gm13443 | 3.7360942 | 1.20E-14 | 1.78E-11 |
| Eno1b* | 2.8526997 | 9.26E-13 | 1.05E-09 |
| Gm10036* | 3.6684231 | 4.14E-12 | 4.19E-09 |
| Gm6166 | 3.577652 | 1.18E-11 | 1.08E-08 |
| Glo1 | 0.6829413 | 3.72E-10 | 3.25E-07 |
| Gm14328* | 5.7912464 | 1.41E-09 | 1.17E-06 |
| Gm7292 | 5.939489 | 2.28E-09 | 1.75E-06 |
| Gm43305 | 1.525871 | 1.35E-08 | 9.30E-06 |
| Gm5805 | 1.0978309 | 1.95E-08 | 1.29E-05 |
| Samd11 | 1.8336422 | 6.14E-08 | 3.47E-05 |
| Gm4735 | 2.061143 | 7.55E-08 | 4.14E-05 |
| 9030025P20Rik | 0.6937218 | 1.50E-07 | 7.78E-05 |
| Ccl25** | 0.9771018 | 1.68E-06 | 0.00070383 |
| Rgs7bp | 0.4717596 | 1.83E-06 | 0.00075022 |
| *Genes have no known function or protein  **Inflammation related genes | | | |

| **Suppl Table 6. Top 20 downregulated genes induced by *Pdgfrβ* *F7* heterozygous mutations in the bAVM of *Eng* EC deleted mice** | | | |
| --- | --- | --- | --- |
| Gene | Log2 fold-change | p-value | Adjst p-value |
| Gm21887* | -2.70056015 | 6.38E-65 | 6.13E-61 |
| Stxbp2 | -1.45390862 | 3.88E-25 | 1.86E-21 |
| G530011O06Rik | -3.42293446 | 2.22E-24 | 7.12E-21 |
| Klf2** | -1.58330182 | 1.10E-18 | 2.63E-15 |
| Fabp5 | -0.87490868 | 2.02E-17 | 4.32E-14 |
| Vill | -3.27027703 | 1.15E-14 | 1.78E-11 |
| CAAA01118383.1 | -1.76809279 | 2.21E-14 | 3.03E-11 |
| Ndufs5 | -0.93434762 | 3.23E-13 | 4.13E-10 |
| Kcnab2 | -0.79867202 | 6.70E-13 | 8.05E-10 |
| Gas5 | -0.82204392 | 1.25E-12 | 1.33E-09 |
| Eps8l1 | -2.20937922 | 6.33E-12 | 6.09E-09 |
| Acaa1a | -0.89636692 | 1.90E-09 | 1.52E-06 |
| Ccl28* | -3.00396421 | 2.40E-09 | 1.77E-06 |
| Eno1 | -0.560052 | 9.44E-09 | 6.72E-06 |
| Vwa1 | -0.83425371 | 2.27E-08 | 1.45E-05 |
| Fcer1g | -1.32408913 | 3.95E-08 | 2.45E-05 |
| S100a9* | -3.4832761 | 5.41E-08 | 3.25E-05 |
| Megf6 | -1.30981827 | 6.00E-08 | 3.47E-05 |
| Zfp36 | -1.54180834 | 9.36E-08 | 5.00E-05 |
| B3galt6 | -0.86222612 | 2.02E-07 | 0.00010226 |
| *Genes related with immune and inflammation  **Anti-inflammation genes | | | |
